# The planktonic stages of the salmon louse (*Lepeophtheirus salmonis)* are tolerant of end-of-century *p*CO_2_ concentrations

**DOI:** 10.7717/peerj.7810

**Published:** 2019-10-14

**Authors:** Cameron R.S. Thompson, David M. Fields, Reidun M. Bjelland, Vera B.S. Chan, Caroline M.F. Durif, Andrew Mount, Jeffrey A. Runge, Steven D. Shema, Anne Berit Skiftesvik, Howard I. Browman

**Affiliations:** 1 Institute of Marine Research, Bergen, Norway; 2 Bigelow Laboratory for Ocean Sciences, East Boothbay, ME, United States of America; 3 Austevoll Research Station, Institute of Marine Research, Storebø, Norway; 4 LEMAR, Institut Français de Recherche pour l’Exploitation de la Mer, UMR 6539 (UBO/CNRS/IRD/Ifremer), Plouzané, France; 5 Department of Biological Sciences, Clemson University, Clemson, SC, United States of America; 6 School of Marine Sciences, University of Maine, Orono, ME, United States of America; 7 Gulf of Maine Research Institute, Portland, ME, United States of America

**Keywords:** Salmon lice, Copepod, Ocean acidification, Parasite, Energetics, Metabolism, Growth, Lipid, *Lepeophtheirus salmonis*, Aquaculture

## Abstract

The copepod *Lepeophtheirus salmonis* is an obligate ectoparasite of salmonids. Salmon lice are major pests in salmon aquaculture and due to its economic impact *Lepeophtheirus salmonis* is one of the most well studied species of marine parasite. However, there is limited understanding of how increased concentration of *p*CO_2_ associated with ocean acidification will impact host-parasite relationships. We investigated the effects of increased *p*CO_2_ on growth and metabolic rates in the planktonic stages, rearing *L. salmonis* from eggs to 12 days post hatch copepodids under three treatment levels: Control (416 µatm), Mid (747 µatm), and High (942 µatm). The *p*CO_2_ treatment had a significant effect on oxygen consumption rate with the High treatment animals exhibiting the greatest respiration. The treatments did not have a significant effect on the other biological endpoints measured (carbon, nitrogen, lipid volume, and fatty acid content). The results indicate that *L. salmonis* have mechanisms to compensate for increased concentration of *p*CO_2_and that populations will be tolerant of projected future ocean acidification scenarios. The work reported here also describes catabolism during the lecithotrophic development of *L. salmonis,* information that is not currently available to parameterize models of dispersal and viability of the planktonic free-living stages.

## Introduction

Predictions of long-term change in *p*CO_2_ and pH in the ocean, and their possible consequences for marine life, have driven intense research activity into the effects of these drivers on marine organisms (e.g., Caldeira & Wickett, 2003; Fabry et al., 2008; Dupont & Pörtner, 2013). Meta-analyses indicate variable responses to *p*CO_2_ among taxa, species within taxa, populations within species, and individuals in any given experiment (e.g., Kroeker et al., 2010; Whiteley, 2011; Garrard et al., 2012; Wittmann & Pörtner, 2013). While this maturing field of research indicates that there will be “winners and losers” in response to ocean acidification (OA), it is more difficult to predict the extent to which future change will impact ecosystems (e.g., Dupont, Dorey & Thorndyke, 2010; Gaylord et al., 2011).

Energy is required to maintain physiological homeostasis in response to environmental change, and responses to environmental stressors are frequently assumed to increase metabolism. However, it is difficult to measure energetic costs directly because organisms can compensate with the reallocation of resources within the organisms’ energy budget (Pan, Applebaum & Manahan, 2015) or by changes in behavior. In addition, the effect of *p*CO_2_ on biological endpoints such as growth is often masked by the saturating food concentrations that are used in most experiments (Ramajo et al., 2016). This study investigates the energetic costs of increased *p*CO_2_ on the lecithotrophic stages of *Lepeophtheirus salmonis* (hereafter referred to as salmon lice).

Salmon lice are obligate ectoparasites which feed on mucus, tissue and blood, causing sores, immunosuppression, and reduced feed conversion efficiency in hosts (Torrissen et al., 2013). Salmon lice infestations on Atlantic salmon (*Salmo salar*) aquaculture cause direct losses and require the implementation of control efforts, which cost an estimated 1 billion USD in economic loses in 2015 (Torrissen et al., 2013; Igboeli, Burka & Fast, 2014; Liu & Bjelland, 2014; Brooker, Skern-Mauritzen & Bron, 2018). They have also been linked to the decline of some wild salmonid populations, which has prompted regulatory restrictions on salmon aquaculture (Krkosek, Lewis & Volpe, 2005; Krkosek et al., 2007; Krkosek et al., 2012; Costello, 2009; Torrissen et al., 2013; Thorstad et al., 2015; Vollset et al., 2017). The importance of salmon lice has motivated several decades of concerted research, making them one of most well-studied species of marine parasite and, thereby, an ideal model species for OA research.

Salmon lice hatch from egg strings produced and carried by females. The salmon louse has 8 developmental stages which are easily differentiated and are characterized by distinct behaviors and ecologies (Hamre et al., 2013; Eichner, Hamre & Nilsen, 2015). During the early developmental stages, prior to host attachment, the louse consumes only the lipid stores contained within the yolk sac. Once the louse metamorphoses into the infective stage (3.81 days post hatch (DPH) at 10 °C), it seeks a salmon host on which it will attach and feed (Samsing et al., 2016). In this study, we exploit the finite energy reserves carried by the non-feeding, free-living life history stages of the salmon louse to investigate the metabolic cost of increased *p*CO_2_. Two instantaneous measures of metabolism (oxygen consumption, and mitochondrial membrane potential) and four metabolic endpoints (carbon, nitrogen, lipid volume, and fatty acids) were measured to determine the impact of increased *p*CO_2_ on the developing planktonic stages.

## Methods

### Experimental design

The experiments were conducted at the Austevoll Research Station, Institute of Marine Research, Norway (60.086N, 5.262E). On the 18th of March 2016, 50 Atlantic Salmon (weight = 300–450 g) were collected from the station’s experimental sea cages and placed in a holding tank with a load of 1-6 female salmon lice per fish. The holding tank was connected to flowing seawater pumped from Bjørnafjord at a depth of 160 m, sand filtered and additionally passed through a 20 mm Arcal disc. As reported by Runge and others (2016), the pH (NBS) of the ambient seawater is 7.95, which corresponds to a *p*CO_2_ of 580 µatm. On May 10th, 14th and 24th, 2016 salmon were sampled using dip nets, anesthetized with tricane methanesulfonate (MS-222), and female sea lice with egg strings were removed using forceps, after which the fish were immediately returned to the holding tank. The salmon remained in the holding tank until the end of the experiment in June 2016 and were then returned to the station’s sea cages. In May 2017, salmon (mean weight of 3.4 kg) were sampled from those same sea cages and additional female lice with egg strings were collected for supplemental measurements of respiration by embryos in egg string.

Egg strings were separated from the females and placed into one of three hatchery tanks under the control experimental conditions (416 µatm *p*CO_2_, 10.5 °C). Within the holding tanks, egg strings were incubated in several hatching chambers made from PVC pipe with a diameter of 10 cm and depth of 15 cm. The top of the chamber was suspended above the water line while the bottom of the chamber was sealed with 80 µm mesh, allowing constant water exchange while preventing animals from escaping. Approximately 31,000 newly hatched salmon lice were transferred from these chambers to the 15 experimental treatment tanks. Following the methodology described by Runge and others (2016), inoculation of treatment tanks was conducted over a period of 8 days beginning on May 14th 2016, with staggered distribution to the three *p*CO_2_ treatment levels. The experiment in each treatment tank lasted for 12 days starting with the inoculation of newly hatched sea lice, and ending with the take down of the tank and sampling of remaining animals for fatty acid analysis. At no time during the experiment were the sea lice provided a food source. Non-feeding nauplii developed to the infective copepodids and were reliant upon lipid reserves until the conclusion of the experiment.

### Experimental conditions

There were three *p*CO_2_ treatment levels: a ‘High’ of 942 µatm representing IPCC (Intergovernmental Panel on Climate Change) worst case scenario end of the century projections, an intermediate ‘Mid’ of 747 µatm and the ‘Control’ of 416 µatm, representing current conditions. Treatment conditions were maintained through the addition of CO_2_ stripped air (in the case of the Control treatment) or CO_2_ enriched seawater that was created by bubbling CO_2_. A full description of the experimental facilities and CO_2_ treatment system can be found in Bailey and others (2016). Daily measurements of tank conditions and analysis of carbonate chemistry were conducted to assess consistency of conditions throughout the experiment. Temperature and salinity in the tanks were measured daily using a hand-held multimeter (Cond 340i conductivity meter: WTW, Germany). The temperature in the experimental tanks increased, from a mean of 10.6 °C at the start of the experiments on May 14th to 10.9 °C at the termination of experiments on June 2nd. Temperature differences were normalized for by calculating degree days: *DD* = [(*T*_*MAX*_ + *T*_*MIN*_)/2] - *T*_*BASE*_, with *T*_*BASE*_ set to 0 °C (McMaster & Wilhelm, 1997). Degree days provide a single continuous variable that normalizes for small differences in temperature between treatments and replicates over the course of the experiment.

Water samples (100 mL) were collected daily from each treatment tank for spectrophotometric measurement of total scale pH using m-cresol purple dye (SOP 6b, Dickson, Sabine & Christian, 2007). Carbonate chemistry was determined independently from routine observations with 40 measures each of total alkalinity, temperature, salinity, and nutrients (phosphate, silicate and nitrate). The samples (250 mL) were fixed with a saturated mercuric chloride solution (Riebesell et al., 2010) and held in the dark at 8 °C until analysis. A detailed description of the protocols carried out for spectrophotometric pH and carbonate chemistry measurements can be found in Bailey and others (2016).

### Biological measurements of catabolism

After hatching, sea lice develop through two naupliar stages, then progress to the infective copepodid stage before attaching to a host (Hamre et al., 2013; Eichner, Hamre & Nilsen, 2015). Since all the free-living planktonic stages are non-feeding, the animals metabolize their lipid reserves and continuously lose mass (Tucker, Sommerville & Wootten, 2000). The biological measurements tracked metabolism throughout this non-feeding period in order to compare catabolism across treatments. Along with the collection of egg strings for analysis, each tank was sampled three times over the 12 day experiment, once while the animals were in the naupliar stage and twice while they were copepodids.

Sampled animals and egg strings were processed for measurement of dry weight, carbon and nitrogen content (C/N); oxygen consumption rates (OCR); mitochondrial activity, and 3D measurement of lipid globule volumes. At the end of the experiment, remaining animals in each treatment tank were collected for analysis of their fatty acid (FA) profiles. The additional egg strings collected in 2017 were analyzed for OCR in ambient seawater (580 µatm *p*CO_2_) at a temperature of 10.5 °C. Respiration rates for egg strings were not observed in 2016 since a single OCR measurement took >8 h to complete and all egg strings were needed immediately for tank inoculations.

**Figure 1:**
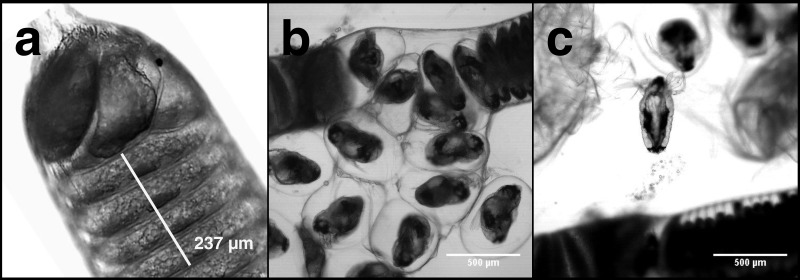
*L. salmonis* naupplii hatching from egg string. The progression of egg hatching is depicted; (A) the terminal section of an egg string with stacked eggs visible; (B) the egg string casing is split and the individual eggs begin to separate from the string; (C) a nauplius newly emerged from an egg.

Carbon and nitrogen content in nauplii and copepodids was assessed by pipetting a known number of animals into an aluminum weighing dish, removing the excess seawater, and drying the sample in an oven at 60 °C until a constant weight was reached. Egg strings were measured intact, but to calculate the dry weight, carbon, and nitrogen content of individual eggs it was necessary to count the number in each egg string. Egg strings are composed of a rigid casing that can contain >500 eggs stacked on top of one another in a regular pattern (Fig. 1). Newly extruded egg strings appear white in color and individual eggs are not easily distinguished initially, but as they develop the pigmentation becomes more pronounced, with pigmentation being greatest just prior to hatching. We examined each egg string under a Leica MS5 dissecting microscope fitted with a Moticam 10 digital camera, with which images were taken. Images were processed using ImageJ (NIH, USA) to count the eggs contained in each string. Excluding the terminal segments where eggs do not develop (Fig. 1A), egg string length was measured using ImageJ’s segmented line tool. Segments of each egg string were measured and the number of eggs within that section were counted. An overall average of eggs per egg string length was then used to calculate the number of eggs in each string. The same procedure was used on the capsules discarded when nauplii hatched from the egg strings. Subtracting the capsule values from the full egg string measurements provided the basis for the per egg dry weight, carbon, and nitrogen content. After imaging, the egg strings were rinsed by dipping them in a sequence of three five mL baths of distilled water. They were then placed in a pre-weighed aluminum dish in an oven at 60 °C and were reweighed after 24 h to the nearest 1 µg on a Mettler-ToledoUMX2 microbalance. All samples were sealed in a vacuum chamber with desiccant and transported to the Darling Marine Center (Walpole, ME, USA) for C:H:N analysis. Samples for C:H:N analysis were combusted in a Perkin Elmer 2400 Series II CHNS/O analyzer equipped with a thermal conductivity detector using ultra high purity helium as a carrier gas.

### Respiration, measured as oxygen consumption

Oxygen consumption rates (OCR) were measured using animals collected from each replicate tank and transferred to the respiration chambers. The mean sample size for OCR was 95.1 animals, consistent with our target sample size of 100, but to reduce handling stress we did not make precise counts of the animals until after the measurements were made, which resulted in a large range of sample sizes (47–237). Supplemental measurements of OCR were made in 2017 on egg strings taken from ambient seawater conditions. Each sample contained 1–3 egg strings which were imaged for egg count after the OCR measurement was taken, giving a range of 307–1,131 eggs per sample.

The respiration chambers were filled with 0.2 µm filtered seawater (FSW) containing no air space (4.3 mL) and closed by a ground glass top equipped with a pinhole (0.4 mm) to accommodate the microelectrode. Dissolved oxygen concentrations were measured using a Clark-type oxygen microelectrode (Unisens; Aarhus, Denmark). Each electrode was calibrated with an anoxic standard of 0.1 M sodium ascorbate and 0.1 M sodium hydroxide solution, and a 100% oxygen saturation point attained through vigorous bubbling of FSW. The FSW also served as the default condition for respiration measurements, with animals and without. All oxygen measurements were made at 10.5 °C (+0.01 °C) in a ThermoScientific water bath (Model A10B with thermostat SC100). Oxygen concentrations within the chambers were measured every 2 s for a minimum of 2 h. The overall oxygen consumption rate for each trial was determined through linear regression of oxygen over time, corrected for changes in control FSW, and not including any points below 80% oxygen saturation.

### Fatty acid analysis

At the end of the experiments (12 DPH), the remaining animals within each treatment tank were sorted to remove debris and transferred into microcentrifuge tubes. Approximately 1,000 animals per sample were required to obtain enough mass to reliably measure. A total of 14 samples of 3–5 egg strings each were rinsed with distilled water, freeze dried, and placed in a −80 °C freezer until transport and analysis. The samples were processed by Bigelow Analytical Services (Bigelow Laboratory for Ocean Sciences, East Boothbay ME). FAs were converted to FA methyl esters (FAMEs) in a one-step extraction direct methanolysis process (Meier et al., 2006) following the procedures detailed in Jacobsen, Grahl-Nielsen & Magnesen (2012). FAMEs were analyzed on a gas chromatograph with mass spectrometric detector (Shimadzu GCMS-QP2010 Ultra; Shimadzu Scientific Instruments, Columbia, MD). Individual FAMEs were identified via comparison to standard mixture peak retention times and fragmentation patterns using the NIST-library of compound mass spectra. FAME concentrations were calculated from 200 peak area relative to that of a C19:0 internal standard that was added to each sample prior to extraction. In preparation for data analysis, the FA data was converted to percentage and normalized with a sqrt arcsine transformation.

### 3D Imaging of lipid volume

**Figure 2:**
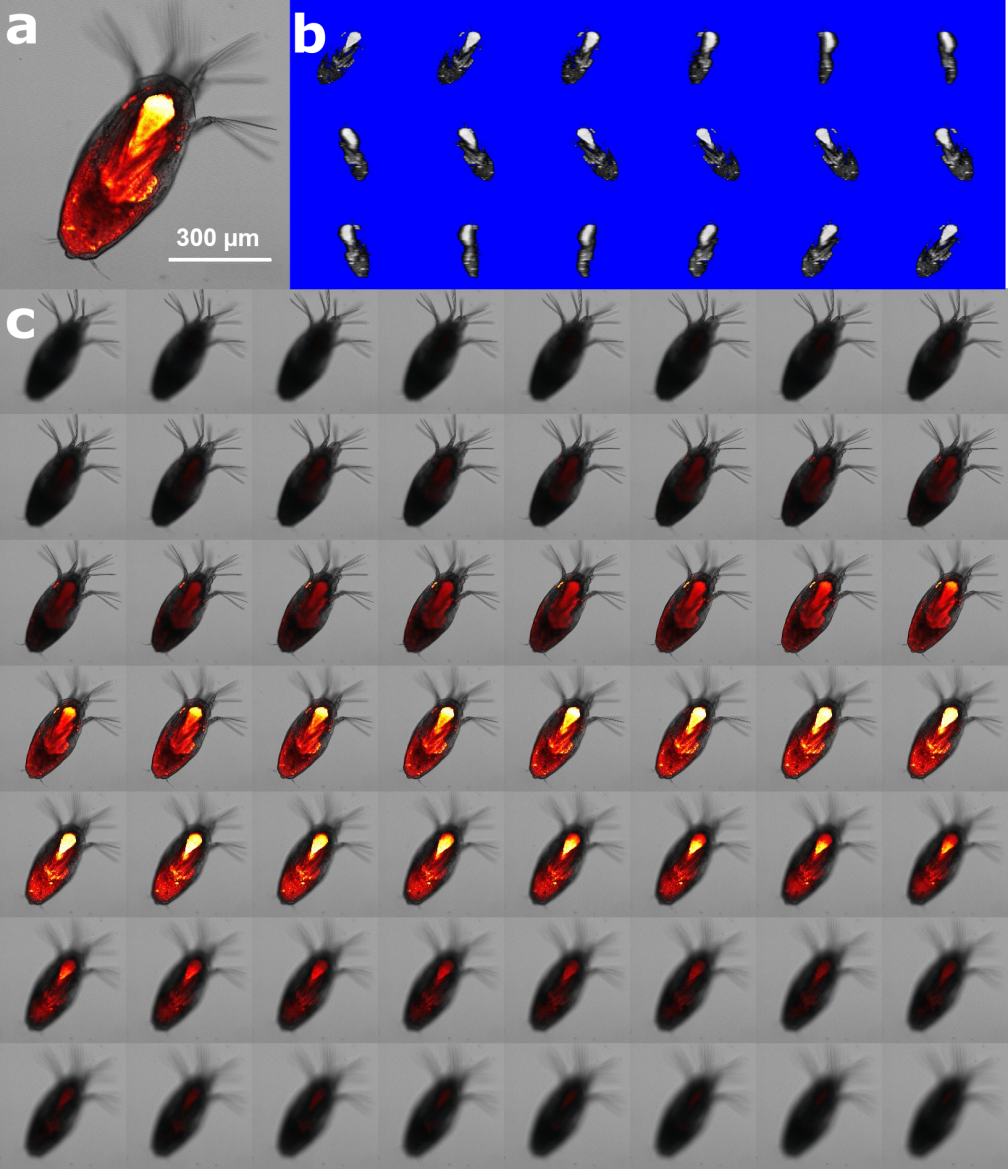
Method of lipid quantification of *L. salmonis* nauplii by nile red (NR) staining. (A) Lipid rich locations bind with NR emitting florescence characterized by confocal microscopy. (B) 3D-reconstruction of the fluorescent signals in white pixels enables 3D visualization of the lipid location in 360° rotation. (C) Z-layers of fluorescent signal beyond the threshold value are shown in grey voxels, the number of voxels was counted and multiplied by calibrated voxel volume (nL) for quantification of the lipid volume.

Lipid volume was observed in 10-20 animals from each replicate on the 2nd and 8th DPH, when lice were in the nauplius and copepodid stages, respectively. The animals were treated with 7.8% w/v MgCl_2_ seawater to sedate them, and were then preserved in 4% paraformaldehyde FSW for microscopy analysis at Clemson University. Animals were stained overnight with 50 µM Nile Red (NR) solution (1mM stock solution prepared in DMSO; stored at 4 °C) in FSW and then gently washed three times with FSW to remove unbound NR. Intracellular lipids were fluorescently stained (*λ* exc/ *λ* em = 552/636 nm) with NR solution and a confocal microscope with a 5×objective and a 561 nm laser was used to capture xyz-images (Fig. 2). Using a low magnification objective with resolution setting of 2,048 × 2,048, 8–10 animals can be observed simultaneously, reducing fluorescence intensity differences due to variation in instrumentation. All animals were imaged on the same day to minimize potential intensity change over time and potential fluctuations in laser performance. Fluorescence caused by the lipid stain was isolated using confocal microscopy and captured during image acquisition. Gray value thresholding was applied to the fluorescent images using ImageJ to select the voxels (pixels in three dimensions), and the total number of voxels were then counted through the z-stack of images for each animal. The lipid volume was calculated from voxel number*voxel size, which was calibrated to nL. Calibration of voxel depths (z-dimension) obtained with a 5x objective was carried out on a quantified volume with a 10x objective in order to improve the accuracy of the voxel size calculation.

### *In vivo* measurement of cellular metabolic state

**Figure 3:**
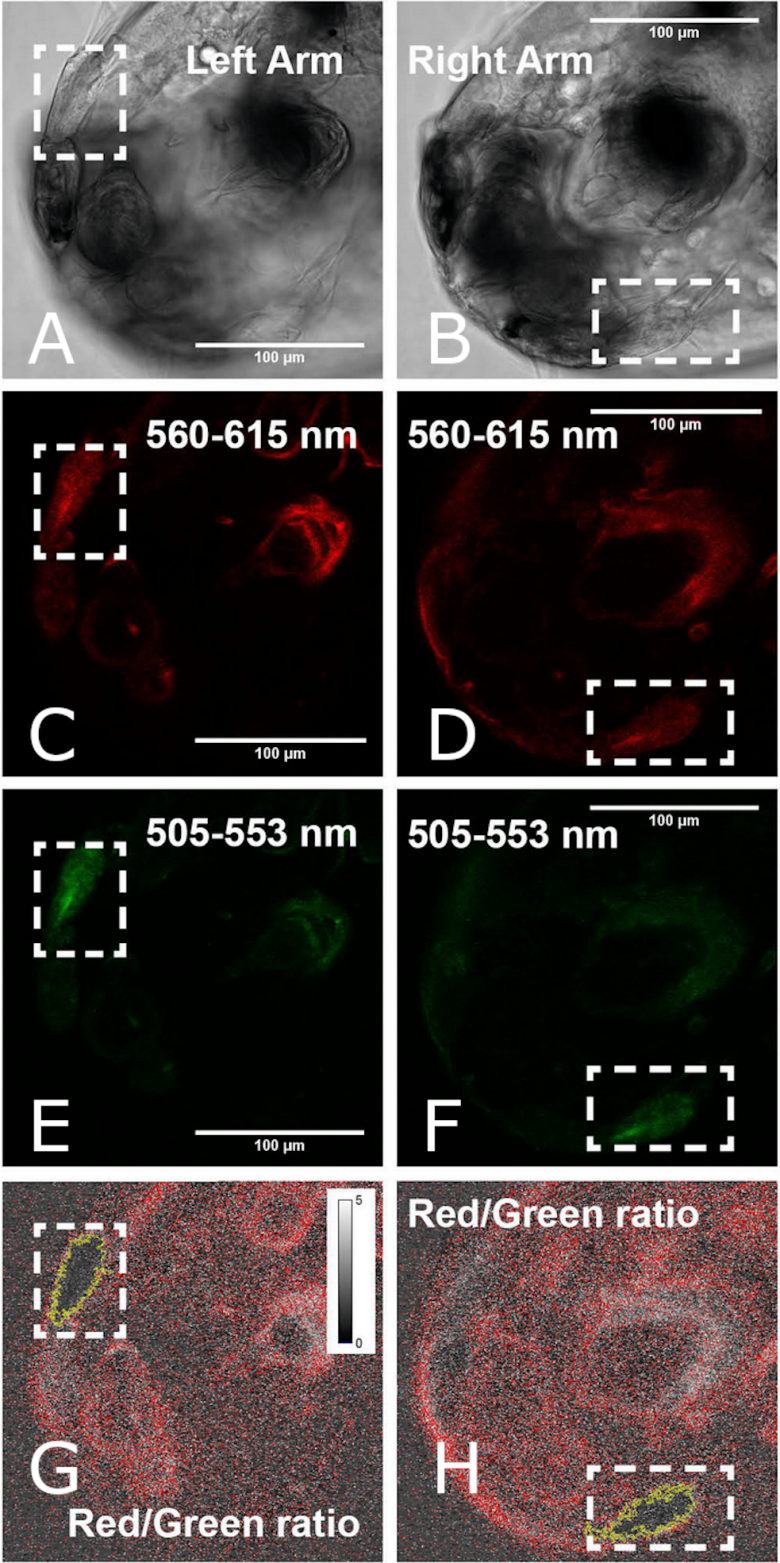
Measurement of mitochondrial activity using image analysis approach in the swimming arms of a 4 DPH *L. salmonis* nauplius. An image representative of the left (left column) and right (right column) arm generates a ratio value of JC-10 fluorescence with respect to the Red (560–615 nm) and Green (505–553 nm) fluorescence. Areas selected (outlined in yellow) were quantified to compare the Red/Green fluorescence ratios.

The metabolic activity of sea lice on 4, 6, 8, and 10 DPH was observed by measuring mitochondrial membrane potential (MMP). Membrane polarization in mitochondria is an indication of active cells. This was visualized by ratiometric measurement of JC-10 (Fig. 3), a fluorescent dye which is reactive to the electrical polarization of membranes (see Park, Chun & Kim, 2013). On each sampling day, the metabolic status of 5-6 living sea lice collected from each replicate tank was analyzed at the Molecular Imaging Center, Bergen University. During transport, animals were kept at low density (five larvae per mL) in two mL sample tubes filled with seawater from the replicate tank leaving minimal head space. At the imaging facility, sea lice were stained with 25 µM JC-10 for 15 min and were then washed with seawater from the experimental tank from which they were collected. Prior to imaging, sea lice were immobilized with molten 2% agarose in FSW; cover slips were then used to support the agarose on the slide. The slide was briefly placed on ice so that the agarose would cure and minimize the lice movement enabling good overlay of the fluorescent channels. Polarized mitochondria were represented by red JC-10 aggregate (*λ* exc/ *λ* em = 488/590 nm) while the green JC-10 monomer (*λ* exc/ *λ* em = 488/525 nm) labeled the presence of mitochondrial membrane. Both the red and green channels were captured on a LSM510 confocal microscope using a 488 nm laser with the same excitation energy (3–15%). Fluorescence images were obtained as 512 × 512 × 16 bit xyz images. The R/G ratio served as a normalized measure of active mitochondria (Park, Chun & Kim, 2013). R/G ratio image was generated for all z layers using the Ratio Plus Plugin in ImageJ, and the area with the best signal was selected using the wand tool with a tolerance value of 0.6.

### Data analysis

Analysis of variance (ANOVA) in R (R Core Team, 2018) was used to determine whether the treatments had any effect on the biological endpoints that were measured. In cases where the data were nonlinear, General Additive Models (GAM) were applied using the -mgcv package (Wood, 2015). In those cases, the GAM smoothed the time term degree day so that a hypothesis test could be performed on the fitted models of differing treatments.

## Results

### Experimental conditions

During the 3-week experiment, mean salinity was 34 PSU and mean temperature was 10.7 °C. A gradual increase in the temperature of the source water led to a difference between treatment tanks of up to ± 0.3 °C. There were no significant differences in degree days between treatments (Table 1). Carbonate chemistry in the treatment tanks was calculated using pH measurements and Alkalinity. The pH was not significantly different between replicate tanks within a treatment but was significantly different between treatments with a pH_T_ of 7.81, 7.60, 7.51 in the Control, Mid and High *p*CO_2_ treatments (Table 1). There were no significant differences in the nutrient concentration or total alkalinity across treatments (Table 1); therefore, the data were pooled to calculate daily carbonate chemistry. The mean *p*CO_2_ levels calculated from the carbonate chemistry for the respective Control, Mid, and High treatments were 416, 747, and 942 µatm_,_ consistent with the target values of 400, 750, and 950 µatm.

**Table 1:** Treatment tank experimental conditions. Mean (±s.e.) Degree Day (base 0 °C), Temperature (°C), pH, Nutrients, and Carbonate Chemistry in treatment tanks for each *p*CO_2_ level and the global average. Total scale pH (**pH**_T,spec_), measured daily spectrophotometrically. Calculations of carbonate chemistry made at spec experimental temperatures using CO2SYS with global mean nutrient concentrations and total alkalinity. Resulting *p*-value from ANOVA of treatment differences, and number of samples is included for each metric.

***p*CO**_2_**treatment**	**Degree day °C**	**Temp °C**	**pH**_T,spec_	**pH**_T,calc_	***p*CO**_2_**µatm**	**HCO**_3_**µmol kg**^−1^	**CO**_3_**µmol kg**^−1^	**CO**_2_**µmol kg**^−1^	**A**_T_**µmol kg**^−1^	**Nitrite µmol L**^−1^	**Nitrate µmol L**^−1^	**Phosphate µmol L**^−1^	**Silicate µmol L**^−1^
Control	136.1 ± 3.5	10.6 ± 0.03	7.81 ± 0.002	8.03 ± 0.002	416 ± 1.7	128.4 ± 0.08	17.9 ± 0.08	2,281 ± 2.5	2,281 ± 2.5	0.04 ± 0.01	4.84 ± 1.47	0.27 ± 0.08	5.65 ± 0.10
Mid	129.5 ± 0.9	10.8 ± 0.05	7.60 ± 0.001	7.80 ± 0.002	747 ± 3.0	81.0 ± 0.22	32.0 ± 0.10	2,281 ± 2.8	2,281 ± 2.8	0.06 ± 0.03	1.93 ± 1.26	0.08 ± 0.03	5.70 ± 0.08
High	128.6 ± 0.6	10.7 ± 0.03	7.51 ± 0.002	7.71 ± 0.002	942 ± 5.2	66.1 ± 0.29	40.7 ± 0.20	2,277 ± 2.0	2,277 ± 2.0	0.04 ± 0.01	1.79 ± 1.05	0.18 ± 0.05	5.59 ± 0.07
Global	131.5 ± 1.5	10.7 ± 0.03	–	–	–	–	–	–	2,280 ± 1.5	0.04 ± 0.01	3.01 ± 0.78	0.19 ± 0.04	5.64 ± 0.05
*P-value*	0.5150	0.0085	<0.001	<0.001	<0.001	<0.001	<0.001	<0.001	0.3808	0.677	0.173	0.176	0.713
*N*	14	202	183	183	183	183	183	183	42	34	34	34	34

### Catabolism of energy stores: carbon, nitrogen, FA, and lipids

**Figure 4:**
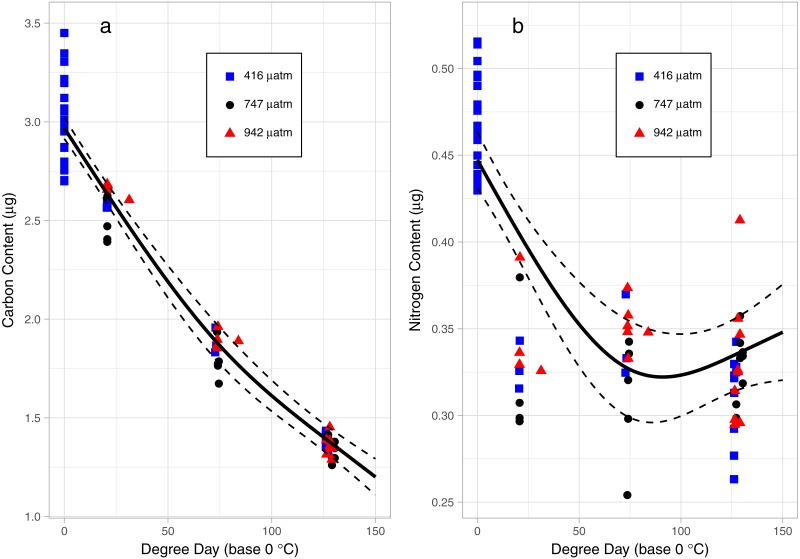
Carbon (A) and Nitrogen (B) content of *L. salmonis* in relation to degree day and *p*CO_2_ treatment level. Egg string observations included at degree day 0. Treatments are Control (416 µatm), Mid (747 µatm), and High (942 µatm). A General Additive Model was fit to the data, the smoothed term with 95% confidence intervals is depicted by the solid and dashed lines.

**Figure 5:**
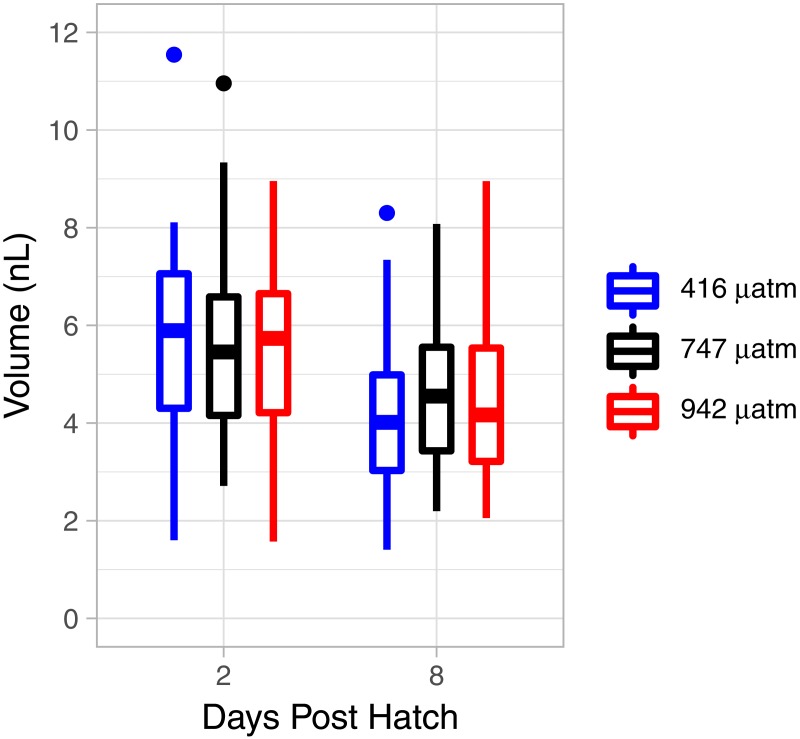
*L. salmonis* lipid volume by age and *p*CO_2_ treatment. Treatments are Control (416 µatm), Mid (747 µatm), and High (942 µatm).

There was a consistent pattern of catabolism with age (DPH) and degree day across all treatments; carbon, nitrogen, lipid volume and fatty acids all declined during the experiment. Image analysis revealed 16.9 ± 0.26 eggs per mm, with a mean of 318 ± 7.9 eggs per string. Mean dry weight of a single salmon louse egg was 5.13 ± 0.082 µg and contained 2.98 ±0.05 µg of carbon and 0.47 ± 0.006 µg of nitrogen. Each egg capsule weighed 0.48 ± 0.035 µg and contained 0.14 ± 0.01 µg of carbon and 0.05 ± 0.002 µg of nitrogen. Carbon and nitrogen content declined as animals developed through naupliar and then copepodid stages (Figs. 4A & 4B). The degree day relationship with both carbon and nitrogen was significant (GAM, *p* < 0.001), but the pattern of catabolism differed. Nitrogen content dropped 30% after eggs hatched, going from 0.47 ± 0.006 µg to 0.328 ± 0.008 µg 2 DPH in the ambient treatment, and then decreasing an additional 5% to 0.311 ± 0.008 µg 12 DPH. Meanwhile carbon content declined in a linear fashion, which fit to the polynomial model: *f* (Carbon µg) = 2.97 – 0.019 degree day + 0.00005 degree day^2^, (adjusted *r*2 = 0.96). The decline in carbon content from 2 DPH (2.578 ± 0.012 µg) to 12 DPH (1.382 ± 0.010 µg) amounted to a 5% reduction per day. Lipid volume in the animals also declined: the mean volume fell from 5.54 ± 0.19 nL ind^−1^ 2 DPH, to 4.42 ± 0.19 nL ind^−1^ 8 DPH, a 3% reduction per day (Fig. 5, Age (DPH): ANOVA *F* (1,171) = 16.34, *P* < 0.001). Total fatty acid content (FA) decreased 6.6% per day from egg strings to 12 DPH copepodid (Table 2).

Of the 36 identified FAs, the 5 most abundant FAs each contributed a minimum of 3% to the total. Those 5 FAs, along with saturated (SFA), monounsaturated (MUFA), and polyunsaturated FA (PUFA), were tested for differences in the proportion between egg strings and 12 DPH copepodids. All FAs tested differed significantly based on stage (Table 2). The greatest proportional change in FA type between egg strings and copepodids occurred in PUFA, which decreased by 56%, while MUFA increased by 38% and SFA increased by 6%. Oleic acid (C18:1n-9) exhibited the greatest proportional reduction from egg to copepodid, decreasing by 93%. The SFA C16:0 and C18:0 had a proportional increase of 40% and 67% respectively, and while the PUFA DHA (C22:6n-3) increased 36%, EPA (C20:5n-3) decreased 6%.

### Instantaneous measures of catabolism: OCR, MMP

**Figure 6:**
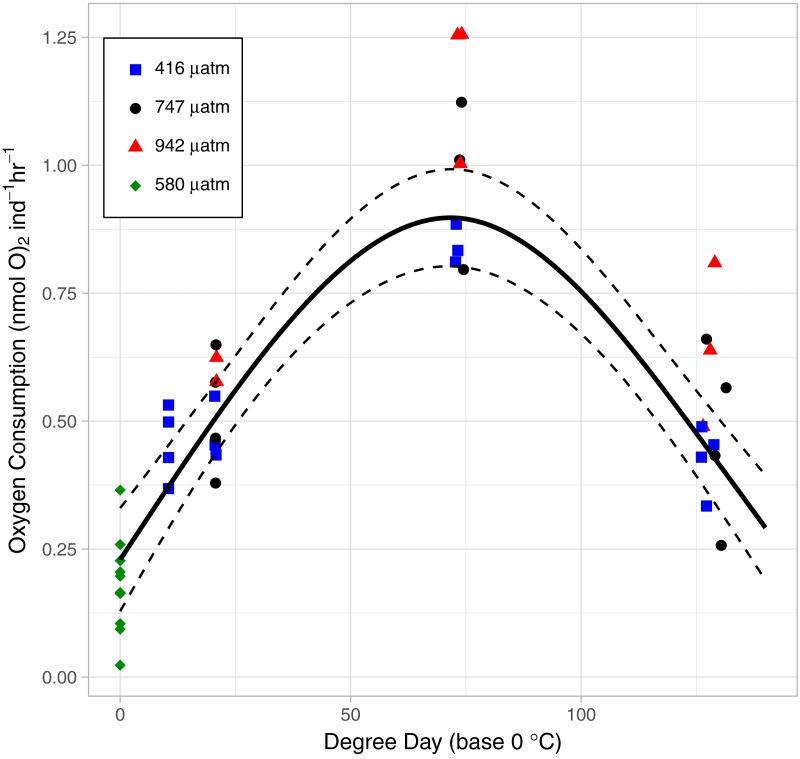
The relationship between Oxygen Consumption Rates and degree days in *L. salmonis* under different *p*CO_2_ treatments. Treatments are Control (416 µatm), Mid (747 µatm), and High (942 µatm). Supplemental egg string measurements were taken at ambient water conditions (580 µatm) and from a separate cohort of animals. Egg string observations are included at degree day 0. A General Additive Model was fit to the data; the smoothed term with 95 % confidence intervals is depicted by the solid and dashed lines.

As the lice hatched and developed to late stage copepodids, respiration followed a pattern of first increasing and then decreasing oxygen consumption rate (OCR) (Fig. 6). Specifically, in the control treatment, OCR increased from 0.479 ± 0.035 nmol hr^−1^ at 2 DPH to 0.843 ± 0.022 nmol hr^−1^ at 7 DPH, and then decrease to 0.427 ± 0.033 nmol hr^−1^12 DPH. The relationship with degree day (*T*_*BASE*_ of 0 °C) was significant for all treatments (GAM, *P* < 0.001, adjusted *r*2 = 0.854, Fig. 6).

**Table 2:** *L. salmonis* fatty acid profiles by stage and treatment. 36 Fatty Acids (FA) were identified, the table columns show the mean weight (ng) of the major FA and the weight of Saturated (SFA), Monounsaturated (MUFA), and Polyunsaturated (PUFA) FA. Profiles are from egg strings and 12 DPH copepodids, which are presented combined and separated by treatment. Treatments are Control (416 µatm), Mid (747 µatm), and High (942 µatm). Sample number and *p*-values from ANOVA are indicated.

**Fatty Acid Profile Components**									
**by Stage**									
****	**C16:0**	**C18:0**	**C18:1n-9**	**C20:5n-3**	**C22:6n-3**	**SFA**	**MUFA**	**PUFA**	**Total**
Egg String (ng)	158.6	30.7	144.1	49.8	253.3	892.0	228.6	252.1	1,372.7
Copepodids (ng)	46.9	10.9	2.2	9.9	73.0	199.7	67.1	23.7	290.6
									
*P-value*	<0.001	<0.001	<0.001	<0.001	<0.001	<0.001	<0.001	<0.001	<0.001
*N*	19	19	19	19	19	19	19	19	19
**by Treatment**									
	**C16:0**	**C18:0**	**C18:1n-9**	**C20:5n-3**	**C22:6n-3**	**SFA**	**MUFA**	**PUFA**	**Total**
Control (ng)	36.7	8.1	1.3	8.4	67.2	165.6	52.6	15.9	234.1
Mid (ng)	52.5	12.3	2.6	10.6	76.9	217.4	75.1	27.5	320.0
High (ng)	50.4	11.8	2.6	10.6	74.5	211.9	72.1	26.8	310.8
									
*P-value*	0.094	0.135	0.152	0.323	0.258	0.090	0.113	0.219	0.120
*N*	10	10	10	10	10	10	10	10	10

**Figure 7:**
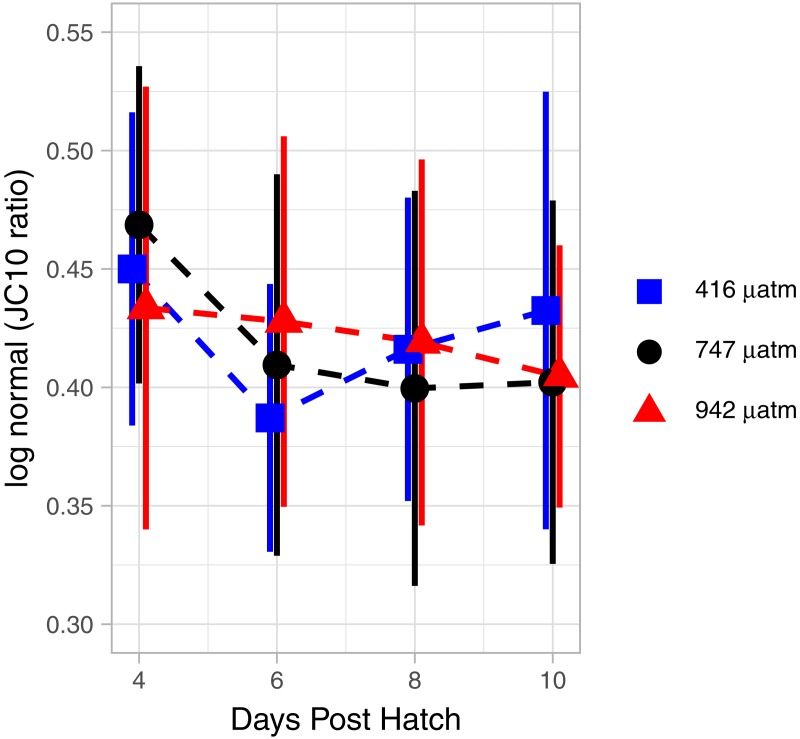
Mitochondrial activity in swimming arm of *L. salmonis* nauplii under *p*CO_2_ treatment. Ratios are log normal, 95% sem error bars are depicted with age offset for visibility. Treatments are Control (416 µatm), Mid (747 µatm), and High (942 µatm).

The mitochondrial activity in the swimming arm, as measured by MMP, was not significantly related to degree day (Fig. 7; GAM, *P* = 0.062, adjusted *r*2 = 0.015).

### Treatment effects of elevated *p*CO_2_

There were no differences in MMP between treatments. Specifically, when analyzed by degree day, there was no significant difference between the Control and Mid (GAM, *P* = 0.873), nor the Control and High (GAM, *P* = 0.952) *p*CO_2_ treatments (Fig. 7).

OCR in the High *p*CO_2_ treatment was significantly higher than in the Control (GAM, *P* <0.001) (Fig. 6). Control *p*CO_2_ had the lowest coefficient of OCR at 0.48 ± 0.03 nmol hr^−1^ followed by Mid with 0.54 ± 0.05 nmol hr^−1^, and High *p*CO_2_ oxygen consumption was greatest with 0.69 ± 0.05 nmol hr^−1^. Since the OCR measurements of egg strings were conducted on a different cohort of animals, we applied a GAM to the dataset without those measurements—when this was done, OCR in the High *p*CO_2_ treatment was still significantly higher than in the Control (GAM, *P* <0.001).

Nitrogen content of the nauplii and copepodids in the Mid treatment was significantly lower than the Control treatment (GAM, *P* = 0.039), with an estimate of 0.348 ± 0.01 compared to 0.376 ± 0.01. Otherwise, the *p*CO_2_ treatment did not affect catabolism of energy stores with no significant difference found between Control and High in nitrogen content (GAM, *P* = 0.655), or carbon content (GAM, *P* = 0.736), and no difference found between Control and Mid treatments in carbon content (GAM, *P* = 0.081). There was also no significant difference in lipid volume between the treatment groups (ANOVA, *F* (2,171) = 0.03, *P* = 0.967). ANOVA was performed on the 5 most abundant FAs along with the 4 FA groupings and total FA to test for differences between treatments; there were no significant differences (Table 2).

## Discussion

### Catabolism of energy stores

As the salmon lice hatched and developed through non-feeding planktonic stages, the carbon and nitrogen content as well as lipid reserves decreased. During the 12 days of post hatch larval development, 54% of carbon mass and 28% of nitrogen mass was consumed. Nitrogen content declined most during the first 2 days after hatching, reflecting the structural changes taking place during development from egg to nauplii. The steady decline in carbon observed was likely due to the metabolism of energy stores in the form of lipids. The decline in lipid volume coincided with a decline in FA weight and an alteration in the FA profile indicative of the preferential catabolism of certain FAs.

The lipid profile of copepods reflects their life history strategy and diet with wax esters preferentially used for long term storage and triacylglycerols for short term demands (Kattner & Hagen, 2009). The lipid profile of salmon lice is principally composed of triacylglycerols with the FA composition reflecting that of their host fish which results in salmon lice from farmed and wild populations having distinct profiles (Tocher et al., 2010). The FA profiles are also dependent on the stage sampled (this study) and the relative changes in specific FAs from egg to 12 DPH copepodids indicate which FAs are utilized for energy during development (Table 2). For example, both PUFA and MUFA levels decreased significantly. Within the MUFA oleic acid declined sharply, indicating that it is an important energy storage reservoir in salmon lice. Meanwhile, the proportional increase in some SFAs indicates those specific FAs were not as readily catabolized.

Declining energy stores prior to host attachment in *L. salmonis* is typical of lecithotrophic development (e.g., Li et al., 2012; Figueiredo et al., 2008; Werbrouck et al., 2016), and the depletion of these energy stores is linked to temperature and mortality (Brooker, Skern-Mauritzen & Bron, 2018). At the conclusion of our 12 day experiment the salmon lice had reached an average of 131.5 degree days. Using the observed relationship between carbon content and age, we can calculate the carbon remaining at the end of a louse’s life when energy reserves are depleted. Taking the endpoint of 150 degree days cited in modeling studies (Asplin, Boxaspen & Sandvik, 2011; Asplin et al., 2014; Johnsen et al., 2014) 1.24 µg of Carbon or 42% of the amount found in eggs, would remain. That decrease highlights the importance of energy reserves to the planktonic stages; any factor affecting the starting amount, or the rate of catabolism, would have an impact on viable lifespan and, thereby, on host infestation success.

### Instantaneous measures of catabolism

*L. salmonis* is lecithotrophic and therefore consumes energy reserves during development. However, the rate of catabolism is not linear with age. OCR was lowest at the egg stage, increased through naupliar stages, and reached a maximum in the infectious copepodid stage (7 DPH) before decreasing to levels similar to that of egg strings. The high respiration during the infectious stage may be related to the increased metabolic cost of detecting and finding a host. However, at least in this experiment, this increase was unrelated to host seeking behavior since no host stimuli were present to activate them. Only in the presence of chemical cues originating from their host will *L. salmonis* engage in host seeking behaviors such as increasing their swimming activity (Mordue & Birkett, 2009). Likewise, a flickering light stimulus simulating the presence of a host fish swimming overhead (and casting a shadow downwards) induces increased swimming speed in infective-stage copepodids (Fields, Skiftesvik & Browman, 2017). Here, mitochondrial activity in the swimming arm was not elevated during the infectious period between 6 and 10 DPH. Therefore, it is unlikely that the increased respiration is related to changes in swimming behavior, which is consistent with previous findings of swimming activity increasing only in the presence of a stimuli.

The OCRs observed on the 12th DPH is consistent with decreased OCR during starvation (Fields et al., 2015). Adopting a low energy strategy could extend the lice’s infective window. Further investigation into this low energy state, as it relates to temperature and host detection, is warranted. *L. salmonis* would provide a useful ecological model for understanding the parasite-host relationship under conditions of stress, and the resulting parameterization would improve forecasts of infection risk.

### Effects of elevated *p*CO_2_

Obligate lecithotrophy provides a unique opportunity to investigate changes in metabolism in response to environmental stressors. Without the ability to compensate for increased metabolic cost through increased ingestion, directly measuring changes in the consumption rate of energy stores provides a direct comparison of metabolic cost under different environmental conditions. In this study we found that end of the century *p*CO_2_ concentrations produced no effect on growth in the planktonic stages of *L. salmonis* after 12 DPH. While nitrogen content in the Mid treatment was significantly different from the control, the High treatment was not, and there were no other treatment effects in any of the other biological endpoints including carbon, lipid volume, and FA content. However, our data did show a non-linear response to *p*CO_2_ in the metabolic rates of salmon lice with OCR increasing in the High treatment. Interestingly, metabolic compensation did not occur through lower mitochondrial activity in the swimming appendages as measured by MMP. In another experiment in which this method of measuring mitochondrial activity was used, sea urchin sperm under elevated *p*CO_2_ responded to the treatment with lower activity in MMP and a 11% decrease in swimming speed (Schlegel et al., 2015). Although measuring MMP provides a high-resolution method of documenting the metabolic status of the swimming arm, further investigation is needed to determine if swimming behavior or host settlement success is affected by elevated *p*CO_2_.

## Conclusion

The lack of a clear linear effect on the salmon louse (*L. salmonis)* from elevated *p*CO_2_ is consistent with other studies on copepods. Runge et al. (2016) concluded that predicted end of the century *p*CO_2_ concentrations have limited effects on copepods. However, in that study and others the animals can compensate for the added metabolic cost associated with elevated *p*CO_2_ by consuming more. Bailey et al. (2016) considered this mechanism as an explanation for the lack of treatment effects on *Calanus glacialis* exposed to elevated *p*CO_2_, but they rejected it while noting that no effect was observed in the non-feeding naupliar stages. Likewise, the animals here are non-feeding and despite the increased metabolic cost of elevated *p*CO_2_ as observed by increased OCR, there was no observable metabolic consequence. Together, these results suggest that these parasitic copepods are able to compensate for the elevated metabolism or that the metabolic cost is extremely small and inconsequential to the development and survival of the copepod.

As an ectoparasite on a highly migratory anadromous fish (*Salmo salar*), the salmon louse and its host have evolved to handle diverse environments. Coastal habitats are often characterized by relatively low pH with carbonate chemistry strongly influenced by upwelling, eutrophication, and river discharge (Feely et al., 2010; Wallace et al., 2014; Salisbury et al., 2008). Salmon lice will inevitably experience a variable environment that features pH regimes far below that predicted from OA. Considering the tolerance of *L. salmonis* to elevated *p*CO_2_ demonstrated here, and their adaptability documented elsewhere (e.g., Aaen et al., 2015; Mennerat et al., 2017; Ljungfeldt et al., 2017), we should expect them to readily adapt to future OA scenarios.

Despite the ubiquity of parasites and their impact on the ecosystem there is a notable lack of investigative work into host-parasite interactions under conditions predicted by OA (MacLeod, 2016). The arms-race between host and parasite could easily be perturbed by a differential response to elevated *p*CO_2_ in the parasite and host. Further work is needed to understand this fundamental ecological relationship, and the salmon louse *Lepeophtheirus salmonis* represents a readily available model organism.

## Additional Information and Declarations

### Data Availability

The following information was supplied regarding data availability: Raw data is available in the Supplemental File and at Zenodo: Thompson, Cameron RS, Fields, David M, Bjelland, Reidun M, Chan, Vera BS, Durif, Caroline MF, Mount, Andrew, …Browman, Howard. (2019). The planktonic stages of the salmon louse (Lepeophtheirus salmonis) are tolerant of end-of-century pCO2 concentrations [Data set]. PeerJ. Zenodo. http://doi.org/10.5281/zenodo.3406147.
